# Prevalence and Determinants of Self-Reported Injuries among Community-Dwelling Older Adults in the Philippines: A 10-Year Pooled Analysis

**DOI:** 10.3390/ijerph17124372

**Published:** 2020-06-18

**Authors:** TJ Robinson Moncatar, Keiko Nakamura, Kathryn Lizbeth Siongco, Mosiur Rahman, Kaoruko Seino

**Affiliations:** 1 Department of Global Health Entrepreneurship, Division of Public Health, Tokyo Medical and Dental University, Tokyo 113-8519, Japan; tjrtmoncatar.ith@tmd.ac.jp (T.R.M.); klsiongco.ith@tmd.ac.jp (K.L.S.); seino.ith@tmd.ac.jp (K.S.); 2 World Health Organization Collaborating Centre for Healthy Cities and Urban Policy Research, Tokyo 113-8519, Japan; 3 Department of Population Science and Human Resource Development, University of Rajshahi, Rajshahi 6205, Bangladesh; swaponru_2000@yahoo.com

**Keywords:** aging, older adults, community-dwelling, injury, pooled analysis, Philippines

## Abstract

Injury among older adults is a serious health concern, but little information is known about it, particularly in developing countries. This study aimed to determine the prevalence of, and examine the socioeconomic, demographic, and health determinants of, self-reported injuries among older Filipinos. Using a pooled data of 21,316 community-dwelling residents aged 60 years or over from three waves of the Philippine National Demographic and Health Survey, multivariate logistic regression analyses were performed to assess the relationship between participants’ characteristics and reports of injuries. The total prevalence of self-reported injuries over a 10-year period was at 1.2%. Older adults with either government or private health insurance were more likely to report experiencing injuries (adjusted odds ratio (AOR) 1.55, 95% confidence interval (CI), 1.14–2.11), regardless of socio-demographic and economic status. In contrast, female older adults were found to be associated with a lower likelihood of self-reported injuries, after adjustment for other variables (AOR 0.69, 95% CI 0.53–0.88). Older adults who attained secondary education or higher also showed a lower likelihood of self-reported injuries (AOR 0.53, 95% CI 0.31–0.92). The proportion of older adults with injuries in the Philippines is still relatively low. However, preventive approaches with a special focus on gender, educational attainment, and health insurance status of older adults are warranted.

## 1. Introduction

The number of older adults in Southeast Asia is continuously increasing at an unprecedented rate, including in the Philippines [1]. By the end of 2030 and 2050, older Filipinos over 60 years of age will constitute 10.3% and 14.3% of its total population, respectively [2]. Although population aging is regarded as a triumph of public health, medical advancements, and economic development over diseases and injuries, the rapid change in demographic age structure poses a serious challenge to the health and socioeconomic development of all nations [3]. As the number of older people rises, several old age-related health problems such as a higher prevalence of non-communicable diseases, functional difficulties, mental illnesses, and other co-morbidities will have a significant impact on quality of life. Thus, they can be viewed as a vulnerable population requiring specific health and social care needs from families, communities, and the government [4].

Older adults are commonly prone to experience injuries due to functional decline related to greater dependence, physical atrophy, loss of bone mineral density, decreased physical activity, dietary calcium deficiency, or, in women, hormonal withdrawal [5]. Injuries are bodily lesions resulting from acute exposure to energy in amounts exceeding physiologic tolerance that is often categorized as an unintentional or an intentional necessitating public health concern, but is deemed neglected [6]. The higher percentage of injuries observed among older adults has contributed to a greater risk of disabling sequelae, morbidity, and mortality in this population. This has led to frequent health facility visits and a longer hospitalization period, resulting in a greater health care burden [6,7,8,9,10].

Particularly, most injuries among older adults have been recorded to occur within the household [10]. Among these, falls were regarded as the most common injury mechanism affecting older adults, followed by being struck or hit, and being crushed, cut, or pierced, while thermal and chemical effects were less common. Fractures were the most frequent consequences of injury [8]. Existing evidence has revealed that the prevalence of domestic accidents among older adults from high-income countries varies from 7.7% in England [11] to 56% in Italy [12]. In Malaysia and Singapore, which are regarded as developed countries in Southeast Asia, home injury prevalence was at 5.8% [9] to 67.9% [13], respectively. The prevalence of all injuries excluding road traffic-related injuries ranged from 1.3% to 9.1% [5,14], while prevalence of bodily injuries excluding falls and road traffic-related injuries ranged from 0.4% to 2.8% in both low- and middle-income countries [15].

In the Philippines, injuries were regarded as the 4th leading cause of mortality across all ages, with 42.3 deaths per 100,000 individuals, wherein the proportion of all deaths classified as injuries quadrupled within a 35-year period, while unintentional injuries were classified as the 5th leading cause of morbidity, with 307.9 accidents per 100,000 population in 1995 [16]. Regarding empirical studies focused on injuries among community-dwelling older adults, the available information was limited and only referred to a specific type of injury and time period. A study covering a small population from a specific setting identified that 53.6% of older Filipinos reported experiencing falls [17], while recently gathered population-based evidence found out that 2.4% of older Filipinos had fractures of the thigh, hip, or pelvis that were likely due to fall injuries. In addition, 1.4% of older adults had fractures in other body parts [18]. Further nationally representative research is needed to investigate the prevalence of common injuries affecting community-dwelling older adults as this is currently less understood.

Identification of the older adult population at risk of injury is also critical for the prevention of complications and for the improvement of their quality of life. This information is required to serve as a basis for the development of national and local strategies that will effectively prevent injuries from happening, reducing untoward negative effects on the wellbeing of older adults and therefore, effectively catering and responding to the health and welfare needs of older adults. Currently, studies from other countries conducted during a single time period has shown that those populations that include older adults aged 75 or over, women, those residing in rural areas, those that are married, those living with other household members, those that are economically disadvantaged, those that are illiterate, those covered by health insurance, and those with a chronic condition were found to be correlated with a higher prevalence of home accidents, falls, or unintentional injuries [9,14,19,20,21]. However, given the context of the Philippines, it is important to determine the specific socioeconomic, demographic, and health characteristics associated with suffering from common injuries among community-dwelling older adults over the years as these factors may present a different meaning and impact in comparison, particularly with developed settings. In addition, available evidence on injuries were mostly from a smaller number of participants and were implemented during a specific survey year. A study utilizing pooled analysis of information across several data collection periods resulting in a larger sample size is warranted and could allow an overall summary and definite conclusions [22].

Moreover, existing evidence on injuries in the Philippines were commonly centered around the general population regarding fireworks, road-traffic, and disaster-related injuries, as well as injury-specific reports derived from hospitals [16,23,24,25]. The few previous local empirical studies among older Filipinos largely focused on perceptions, quality of life, and older adults in the workforce [26]. Epidemiological studies on the prevalence of domestic injuries among older adults and associated determinants are needed, as existing studies focusing on this population are lacking, particularly within developing countries [9] such as the Philippines. A detailed and comprehensive analysis will render precise and valid information resulting in a clearer understanding of the link between socioeconomic, demographic, and health characteristics on the prevalence of injuries among community-dwelling older adults, which is therefore essential in policy development and implementation.

In this study, we intended to further this important area of inquiry by (1) estimating the prevalence of injuries; (2) identifying the patterns of self-reported injuries according to socioeconomic, demographic, and health characteristics; and (3) determining the characteristics associated with self-reported injuries among community-dwelling older adult populations in the Philippines over a 10-year period using a nationally representative sample.

## 2. Materials and Methods

### 2.1. Data Sources

This study was performed using data from the Philippine National Demographic and Health Survey (PNDHS) conducted for the years of 2008, 2013, and 2017. The PNDHS is a nationally representative, cross-sectional household-based survey conducted every 5 years by the Philippine Statistics Authority (PSA) through the technical support of the Monitoring and Evaluation to Assess and Use Results Demographic and Health Surveys (MEASURE DHS) program of ICF International. The PNDHS sample was drawn from adult Filipinos residing in private residences to obtain information regarding population health and nutrition to be used in evaluating and designing policies, programs, and strategies for improving health services in the Philippines. This survey was also done to ensure comparability across countries and times through the use of standardized measurement tools and techniques including pre-testing, an identical primary questionnaire, extensive survey procedures, interviewer training, and data-processing guidelines.

### 2.2. Study Design, Sampling Size, and Sampling Technique

The 2008 PNDHS employed a stratified three-stage sampling design, while both the 2013 and 2017 PNDHS followed a stratified two-stage sampling design representing 17 regions in the Philippines including urban/rural stratum and clustering by barangays (primary sampling unit or PSU). In addition, this design used the Census of Population and Housing as a frame to ensure population representativeness. A sample of 12,469 households covering a total of 57,629 persons were randomly selected and interviewed from 994 PSUs with a response rate of 99% for 2008. A total of 14,804 households were successfully surveyed composed of 71,893 individuals, yielding a household response rate of 99.4% for 2013, while a total of 27,496 households with 111,643 individuals were randomly chosen and interviewed from 1250 PSUs with a response rate of 99% for 2017. Full survey details pertaining to the sample design and procedures are available online [27,28,29].

Out of the total household members identified in the three survey periods, information from older adults only aged 60 years or older were included in the current study. Older adults with missing information were excluded in the analyses. The number of older adults within the 10-year period used in this study was merged with a total of 21,316. The disaggregated number of older adults per year of survey, wherein separate analyses were also performed, were as follows: 2008, *n* = 4380; 2013, *n* = 5567; and 2018, *n* = 11,369.

### 2.3. Data Collection

The PNDHS utilized three types of questionnaires and the information collected from the Household Questionnaire were utilized in this study, which identified socioeconomic and demographic characteristics, as well as health insurance coverage, health status, and health facility use of each household member. The development of this questionnaire, based on MEASURE DHS model questionnaires, underwent a series of consultative meetings among the various public and private agencies involved. The questionnaires were translated from English into the six major languages used in the Philippines.

### 2.4. Measurement of Variables

#### 2.4.1. Outcome Variable

This study mainly measured self-reported injuries within a 10-year period, as well as in 2008, 2013, and 2017. In the PNDHS, self-reported injuries were determined through the measurement of the prevalence of common injuries affecting community-dwelling older adults. Respondents were asked if they had experienced injuries due to cuts/wounds, burns, or fracture/broken bones in the last 30 days. Those who were reported as having one or more of these conditions were grouped as having injuries and dichotomized as yes = 1, and no = 0.

#### 2.4.2. Exposure Variables

To further elucidate the patterns and particularly the characteristics associated with self-reported injuries among older adults, several socioeconomic and demographic variables, as well as health characteristics, that have been theoretically and empirically linked with experience of having injuries, were included [5,9,19,20,21,30]. Participant’s age was categorized as follows: 60–69, 70–79, and 80 years or over. Gender was labelled as male or female. The living arrangements of older adults were grouped as to whether they were living alone or living with others in the household (one or more adults or children). The type of place of residence was classified as rural or urban in reference to the government classification of barangays or communities. The 17 regions in the Philippines were grouped into three main island groups given the country’s archipelagic attribute: Luzon (Regions 1–5 including Metro Manila, and the Cordillera Administrative Region); Visayas (Regions 6–8); and Mindanao (Regions 9–13 including Autonomous Region in Muslim Mindanao).

Two measures of socioeconomic status were included in this study: education and economic status. Highest educational attainment was based on the Philippine’s formal education system and categorized as having no education or only pre-school, primary or elementary education, or attaining secondary education or higher (i.e., college/university, post-graduate degrees). The wealth index used as a proxy indicator for economic status of older adults classified as poor, middle, or rich, was constructed from data on household assets including ownership of durable goods and housing characteristics. Each asset was assigned a weight (factor score) derived through principal component analysis. Individual households were given a score per asset, and scores were summed by household. The sample was divided into population quintiles wherein each was designated a rank and individuals were ranked according to the total score of the household where they reside.

Health characteristics such as the possession of health insurance (provided privately or by the government) was categorized as yes or no. In addition, reports of having diabetes mellitus, cancer (all forms), or hypertension in the last 30 days were measured in this study. Those with any one or more of these conditions were grouped as having non-communicable diseases or none.

### 2.5. Statistical Analyses

All analyses were performed using SPSS version 23.0 (SPSS Inc; Chicago, IL, USA). Prevalence estimates for reported experience of injury in the last 30 days among community-dwelling older adults by socioeconomic, demographic, and health characteristics were calculated. Differences between these characteristics in reports of injuries were assessed by Chi-square test (χ 2); significance for all analyses were set at *p* < 0.05, two-sided. Multivariate logistic regression was performed, fitting the data to model the crude associations between socioeconomic, demographic, and health characteristics and the defined outcome variable. This was followed by entering all the characteristics simultaneously into the multivariate regression models to determine how the addition of other variables affected the relationship between the outcome variable and each determinant. We estimated the odds ratios (ORs) to assess the strength of the association, and used 95% confidence intervals (CIs) for significance testing. The multi-collinearity of the variables was checked by examining the variance inflation factor (VIF), which was <2.0. All estimates were weighted to correct for non-response and disproportionate sampling based on the complex design of the PNDHS.

### 2.6. Ethical Considerations

The Institutional Review Board of ICF International (Calverton, Maryland, USA) and an ethics panel in the Philippines reviewed all protocols of the three PNDHSs [31]. Permission to use the publicly available online data for this study was requested and approved by the DHS Program, with all identifying information anonymized. Therefore, the present study is exempted from ethical clearance approval. The respondents’ consent for participation was obtained after the survey purpose was described and it was assured that all information provided would be kept confidential, that participation was voluntary, and that participants could refuse to answer any questions or discontinue the interview at any point.

## 3. Results

### 3.1. Prevalence of Self-Reported Injuries among Older Adults

The total prevalence rate of self-reported injuries among community-dwelling older Filipinos over a 10-year period was at 1.2%. Furthermore, as shown in Table 1, the rates of self-reported injuries slightly increased from 1.0% in 2008 and 2013 to 1.4% in 2017.

### 3.2. Differential on Self-Reported Injuries with Characteristics of Older Filipinos

The bivariate analyses revealed several significant differences in the prevalence of self-reported injuries across various socioeconomic, demographic, and health characteristics of community-dwelling older Filipinos (Table 1). Specifically, during the 10-year period, reports of injuries was significantly more common among male older adults than females. Reports of wounds, burns, or fractures in the last 30 days were significantly more frequent among older adults residing in rural areas, those that had no education or pre-school education only, and those who had health insurance. In addition, self-reported injuries were less common among older adults belonging to higher economic bands of wealth.

Higher prevalence of self-reported injuries, particularly in 2017, were statistically significant among older adults aged 70–79 years old, males, and those residing in rural areas. In addition, older adults of a higher economic status reported the experience of injuries significantly less frequently than older adults from low or middle economic bands of wealth. However, in 2008 and 2013, there were no significant differences on reports of injuries among community-dwelling older adults.

### 3.3. Determinants Associated with Self-Reported Injuries among Older Filipinos

Table 2 shows the association between socioeconomic, demographic, and health characteristics and self-reported injuries among community-dwelling older adults. During the 10-year period, in both the crude and adjusted models, older adults possessing health insurance had greater odds of reporting or experiencing an injury (crude odds ratio (COR) 1.39, 95% CI 1.03–1.88; AOR 1.55, 95% CI 1.14–2.11). In contrast, female older adults were negatively associated with report of injuries as compared to males (COR 0.68, 95% CI 0.53–0.87; AOR 0.69, 95% CI 0.53–0.88). This result was also similar among those older adults who attained high school education or higher (COR 0.55, 95% CI 0.34–0.90; AOR 0.53, 95% CI 0.31–0.92) as compared to those who had elementary education only or no education at all.

Per year of survey, in 2017, older adult females were also less likely to report having an injury in both models (COR 0.50, 95% CI 0.36–0.69; AOR 0.48, 95% CI 0.35–0.67), while those belonging to middle economic bands of wealth had higher odds of reporting injury (COR 1.66, 95% CI 1.12–2.46; AOR 1.85, 95% CI 1.22–2.81). In addition, in the adjusted model, older adults residing in the Mindanao Islands and those who attained secondary education or higher were less likely to report experiencing injuries, while this was in contrast to older adults covered with health insurance. 

In 2008, older adults with a higher economic status were negatively associated with reports of injuries in both models (COR 0.50, 95% CI 0.26–0.99; AOR 0.35, 95% CI 0.15–0.82), while those older adults covered with either government or private health insurance were positively associated with reports of injuries after adjustments with other variables. There were no significant associations on self-reported injuries with any socioeconomic, demographic, or health variables in both crude and adjusted models in 2013.

## 4. Discussion

### 4.1. Prevalence of Self-Reported Injuries among Older Adults

Findings from this study using three waves of a population-based representative survey to quantify the larger and accurate status of the prevalence of reported injuries among community-dwelling older adults in the Philippines, during a 10-year period, was determined. Although the total proportion of older adults who reported experiencing injuries was low (1.2%), a marginal increase of cases was observed between 2008–2017. This total percentage was about the same level as that of the average proportion of older adults, who experienced all injuries except road-traffic accidents in five low- and middle-income countries such as South Africa (1.3%), Russia (3.3%), Mexico (4.2%), China (5.1%), Ghana (5.7%), and India (9.1%) [14]. This prevalence, though relatively lower, may suggest that if not commonly happening within communities as compared with communicable and noncommunicable diseases, older adults might not take the occurrence of injuries seriously or recall its existence. This was similar to a study in the United States of America identifying that fall-related injuries were significantly underreported among older adults [32]. Nevertheless, the identified prevalence estimates afflicting older adults emphasizes that the government of the Philippines still needs to place more importance on the predicament of its older adult citizens, because the rate of injuries are expected to further increase as the aging population continues to rise; this prevalence reflects the general well-being of this population and its need for applicable and sustainable preventive approaches.

### 4.2. Differential on Self-Reported Injuries with Characteristics of Older Filipinos

The results of this study also revealed the summary profile of community-dwelling older adults who commonly reported experiencing injuries over a 10-year period. These older adults who were the most affected were males, those residing in rural areas of the Philippines, and educationally disadvantaged persons, as they had not received any formal education. Hence, injuries among older adults with a higher socioeconomic status were less frequently observed. Injury prevention and management must be particularly focused among this segment of older adults in the community. In addition, individuals covered with either government or private health insurance showed a higher prevalence of reports of experiencing injuries.

In previous evidence from Vietnam and Malaysia, females were more commonly identified as being likely to experience injuries as compared with males [9,33]. The result of the present study is in contrast to this, as older females in the Philippines are normally expected to be within their domicile continually receiving care, attention, and support from other household members. This results in a lesser likelihood of having injuries, as compared with male older adults who are still active in the labor force, trying to financially assist with the daily needs of their family. Older Filipinos who have a higher socioeconomic status and a higher educational attainment would report better quality of life [26]. Those experiencing socioeconomic difficulties were more likely to be prone to household hazards or occupational risks as most, despite their age, might still be performing manual labor, which is consistent with a study in Turkey that revealed how low-income individuals had a higher risk of injury at home [34]. Moreover, evidence from Indonesia recognized that older men with poorer socioeconomic backgrounds frequently experienced multiple fall-related injuries [19]. Household structures in the Philippines are steadily changing [35]. Migration among the predominantly young workforce has reduced the number of individuals rendering care assistance and attention to older adult household members left in rural areas, increasing their risk of having an accident within their homes, which is comparable to a study from China [21]. In addition, health insurance coverage among older Filipinos has increased over the years, enhancing the seeking of health care and an awareness with conditions, including injuries. It was also similarly identified, in recently gathered local evidence, that older adults covered with government health insurance often reported illness and utilized outpatient and inpatient care services frequently [36].

Per survey period, this study was not able to observe significant differences on the reporting of injuries among older adults in 2008 and 2013. However, in 2017, aside from gender, place of residence, and socioeconomic status, older adults aged 70–79 years significantly reported experiencing injuries. This result was in contrast to a previous study in Spain wherein those aged ≥85 years had a five-fold greater risk of experiencing injuries than those aged 65–69 years [37]. This is potentially due to better functional ability of older adults in this age group, as most of them possibly retired recently from the labor workforce, thus, still enhancing their active role in performing key activities within their households, increasing their risk of having an injury.

### 4.3. Determinants Associated with Self-Reported Injuries among Older Filipinos

Although the particular profile of community-dwelling older adults frequently experiencing injuries was observed, the specific factors associated with it after consideration of other confounding variables affecting the relationship between outcomes and each determinant was also established. This study highlighted the valuable impact of health insurance coverage among older Filipinos, as this population is mandatorily covered as specified in the law [38], due to its strong association with reports of injury over the years. As mentioned, injuries such as falls are possibly underreported among older adults [32], and it can be inferred from the current study that the greater odds of reporting injuries demonstrated the positive effects of health insurance in raising health awareness by ensuring the elimination of barriers towards equitable access to formal care [39]. This was similarly identified from a previous study on traumatic injuries, where a lack of health insurance had adverse consequences on the utilization of hospital resources impacting health [40]. The Philippine government must further strengthen its information drive and enrollment assistance to cover all older adults in the national health insurance program.

This study also indicated that female older adults had a lower likelihood of reporting injuries, regardless of the effects of other factors. This result is in contrast to evidence from China, which has identified that female older adults were more likely to suffer from unintentional injuries [21]. The gender differences on reports of injuries can be explained by the patriarchal system in the Philippines. Males are the breadwinners, while females are expected to perform traditional roles as mothers, wives, and housekeepers [41]. Due to strong filial and intergenerational support within families, older Filipinos are providing monetary and non-monetary support within their households [42]. Hence, males are opting to continue working or performing labor-intensive household tasks despite their age, increasing their risk of injury as compared to females who are more likely to be in charge of cleaning, cooking, or taking care of grandchildren.

This study also observed that those who had attained at least secondary education or higher were negatively associated with reports of injuries. This conferred the protective impact of receiving higher formal education not just on the economic aspect of individuals, but also on their health and wellbeing. Evidence from Iran similarly identified that older persons with a college or university education were more than 50% less likely to report fall-related injuries than illiterate persons [43]. Individuals with primary or no education at all were the most financially vulnerable group [44]. In the Philippines, high poverty levels are strongly linked with low educational attainment [45]. Poor families are made up of a large number of household members residing in badly constructed shelters with a small floor area, outer walls and roofs constructed from light materials, and floors made of wooden planks or cement [46]. These structural deficiencies in housing may lead to fatal and non-fatal injuries among its settlers, necessitating housing improvements [47]. Moreover, the economy of the Philippines has benefited from the employment of older adults [26], but almost 67% of poor Filipinos work in agriculture and forestry, fishing, and construction [45], which include probable injury hazards.

The determinants associated with the reports of injuries were shown in 2008 and 2017 only, as no significant association was observed in 2013. This suggests that the impact of specific events during this period contributed to the strong relationship between variables. In 2017, aside from other variables with an observed association on the report of injuries in the pooled analysis, older adults from the Mindanao Islands had lower odds of experiencing injuries. This is considerably due to the reason that the data-collection period of PNDHS 2017 was conducted from August to October, which is the peak of tropical cyclone season in the Philippines and mostly affected the Luzon archipelago [48,49]. Aside from damages on infrastructure, natural hazards have negative health consequences among older adults, such as on the occurrence of minor injuries (i.e., open wounds, bruises, and burns) or fractures [50,51]. On the other hand, older adults belonging to middle economic bands of wealth showed a higher likelihood to report injuries in 2017. This result highlighted the health effects on older adults of the growing middle-income families in the Philippines due to the rising number of overseas Filipino workers (OFWs), a group regarded as the third largest recipient of personal remittances in absolute terms in the world, creating a culture of economic dependence [52,53]. Middle-income households rely on cash remittances but may have affected older adult members in performing their daily activities due to the reduced number or absence of individuals rendering assistance, increasing their exposure to injury. In 2008, while the presence of health insurance revealed a positive association, older adults belonging to rich economic bands of wealth showed a negative association in experiencing injury. This finding presented the higher exposure of poor older people due to a lack of resources and capacity to adapt to potential risks. Inequality in the Philippines is among the highest in the world and during this period, not less than 3.8% of the population were severely deprived, resulting in significant adverse health outcomes [54].

In the context of a low- and middle-income country, the results of this study highlighted how the distinct socioeconomic, demographic, and health characteristics of community-dwelling older Filipinos correlated with reports of injuries over a 10-year period. This implies close attention, monitoring, assistance, and targeted approaches with these factors to mitigate the expected impact of injuries among older adults in the future. Prioritization and multi-sectoral efforts towards older adults are necessary to be implemented at the national, regional, and local levels to cater for the characteristics and needs of older adults. In particular, immediate intervention such as the profiling of older adults through inter-sectoral collaboration and the sharing of information between cities or municipal senior citizens’ affairs offices, social welfare and development offices, and health offices should be conducted to identify individuals at risk based on the results of this study and render necessary interventions to prevent the untoward impact of injuries among older adults. In addition, this study notified families of potential repercussions of specific characteristics of older family members, particularly its influence on their risk of experiencing injuries necessitating home modifications or precautions.

### 4.4. Strengths and Limitations of the Study

The strengths and limitations of this study must be taken into consideration when interpreting the results. The primary strength of this study is that it utilized three waves of a representative, randomly selected population-based sample with a high response rate and reliable results. A national sample of older adults for each of the survey years were collected and merged, resulting in valid and precise conclusions regarding the outcome variables and factors associated. Given the limited information on injuries concerning older adults, to the best of our knowledge, this is the first study to observe a larger sample size of older Filipinos from a longer time period to depict a precise picture of injuries for this population. All the three surveys adhered to carefully developed standardized tools to ensure accurate information from the participants. The data collection process for all survey years included robust data quality checks and interviewers were trained extensively for standardized administration of the interview process.

However, some caveats to the findings of this study should be noted. The PNDHS is a cross-sectional study and no causal relationship can be inferred. Information pertaining to injuries were also self-reported and subject to recall bias. Further studies with complex designs are necessary to be conducted to determine any associations between variables and its causality. In addition, the specific types of injuries covered in the three surveys were limited to cuts, wounds, burns, and fractures. Moreover, there was a potential lack of awareness from interview participants on the definition of what is classified as an injury. These limitations might have underestimated the actual prevalence of injuries necessitating an accurate listing and description of what constitutes potential injuries commonly afflicted among older adults in the community. Information regarding the specific setting (i.e., home, community, workplace) on where the injury occurred, including the current occupation, and household roles of older adults, were also not available in the dataset. In contrast with injury-related studies conducted or based in hospital reports, the anatomical location, severity of injury, existing disability among older adults, previous history of injury, health behavior, and other valuable clinical markers were not collected in the PNDHS. The presence of this information would further enrich the study and allow a clearer interpretation of the findings.

## 5. Conclusions

Based on pooled information over a 10-year period of a large, nationally representative sample, the present study revealed that the prevalence of injury among community-dwelling older Filipinos is still relatively low, but marginally increased over the years. In the context of a developing country, this study highlighted several key determinants strongly associated with reports of injury among older adults such as gender, educational attainment, and presence of health insurance. These results, which provided the status and factors correlated with reports of injuries among older Filipinos over a 10-year period, will largely assist policy makers, program implementers, as well as geriatric care workers to identify individuals at risk of injury. Therefore, the study helps design tailor-fitted policies and the implementation of injury-related approaches based on the idiosyncrasies of older persons to prevent or reduce the untoward impact of injury in the community.

## Figures and Tables

**Table 1:** Descriptive statistics according to self-reported injury among older adults in the Philippines, 2008–2017.

Characteristics	Self-Reported Injury ^a^							
	Pooled (*n* = 21316)		2008 (*n* = 4380)		2013 (*n* = 5567)		2017 (*n* = 11369)	
	Yes (*n*)	%	Yes (*n*)	%	Yes (*n*)	%	Yes (*n*)	%
**Prevalence**	266	1.2	46	1.0	58	1.0	162	1.4
**Socioeconomic and Demographic Characteristics**								
**Age (yrs)**								
60–69	154	1.1	27	1.0	31	0.8	96	1.4
70–79	88	1.5	13	1.0	21	1.3	54	1.8
80+	24	1.0	6	1.4	6	1.2	12	0.8
*p* Value		0.074		0.614		0.283		0.041
**Gender**								
Male	142	1.5	19	1.0	26	0.9	97	2.0
Female	124	1.0	27	1.1	32	1.0	65	1.0
*p* Value		0.002		0.868		0.720		<0.001
**Living Arrangements**								
Living with Others	246	1.2	43	1.0	55	1.0	148	1.4
Living Alone	20	1.2	3	1.2	3	1.1	14	1.3
*p* Value		0.995		0.733		0.844		0.670
**Place of Residence**								
Urban	80	1.0	21	1.0	16	0.7	43	1.1
Rural	186	1.4	25	1.0	42	1.2	19	1.7
*p* Value		0.008		0.944		0.068		0.016
**Island Group of Residence**								
Luzon	136	1.2	20	0.9	28	0.8	88	1.5
Visayas	71	1.5	12	1.2	14	1.3	45	1.7
Mindanao	59	1.1	14	1.2	16	1.2	29	1.0
*p* Value		0.228		0.529		0.339		0.164
**Highest Educational Attainment**								
No Education, Preschool	18	1.9	6	1.5	6	2.0	6	2.1
Primary	146	1.3	25	1.0	32	1.0	89	1.6
Secondary or Higher	102	1.0	15	1.0	20	0.8	67	1.2
*p* Value		0.038		0.656		0.127		0.091
**Economic Status**								
Poor	116	1.4	25	1.4	26	1.2	65	1.4
Middle	63	1.7	9	1.1	12	1.1	42	2.3
Rich	87	0.9	12	0.7	20	0.8	55	1.1
*p* Value		<0.001		0.144		0.333		<0.001
**Health Characteristics**								
**Presence of Health Insurance**								
No	61	1.0	21	0.8	25	1.2	15	0.8
Yes	205	1.3	25	1.3	33	0.9	147	1.5
*p* Value		0.033		0.137		0.273		0.061
**Self-Reported NCDs ^b^**								
No	245	1.2	46	1.1	51	0.9	148	1.4
Yes	21	1.2	0	0.0	7	1.2	14	1.5
*p* Value		0.831		0.079		0.514		0.909

*p* values refer to differences between groups; numbers are unweighted; percentages are weighted; ^a^ injury includes wounds, burns, or fractures; ^b^ non-communicable disease (NCDs) includes diabetes mellitus, cancer (all forms), or hypertension.

**Table 2:** Odds ratios for association between socioeconomic, demographic, and health characteristics and self-reported injuries among older adults, Philippines, 2008–2017.

Characteristics	Self-Reported Injury ^a^							
	Pooled		2008		2013		2017	
	COR (95% CIs)	AOR (95% CIs)	COR (95% CIs)	AOR (95% CIs)	COR (95% CIs)	AOR (95% CIs)	COR (95% CIs)	AOR (95% CIs)
**Age (yrs)**								
60–69	1.00	1.00	1.00	1.00	1.00	1.00	1.00	1.00
70–79	1.33 (1.02–1.74) *	1.29 (0.98–1.69)	1.00 (0.50–1.99)	1.02 (0.51–2.05)	1.62 (0.91–2.88)	1.50 (0.84–2.70)	1.34 (0.95–1.88)	1.26 (0.89–1.79)
80+	0.90 (0.58–1.40)	0.86 (0.55–1.35)	1.50 (0.65–3.47)	1.59 (0.66–3.79)	1.33 (0.56–3.14)	1.13 (0.47–2.74)	0.60 (0.31–1.17)	0.55 (0.28–1.08)
**Gender**								
Male	1.00	1.00	1.00	1.00	1.00	1.00	1.00	1.00
Female	0.68 (0.53–0.87) **	0.69 (0.53–0.88) **	1.08 (0.59–1.96)	1.16 (0.64–2.13)	1.12 (0.65–1.93)	1.09 (0.63–1.89)	0.50 (0.36–0.69) **	0.48 (0.35–0.67) ***
**Living Arrangements**								
Living with others	1.00	1.00	1.00	1.00	1.00	1.00	1.00	1.00
Living Alone	1.00 (0.61–1.64)	0.96 (0.58–1.58)	1.11 (0.32–3.82)	1.05 (0.30–3.69)	1.16 (0.37–3.65)	0.99 (0.31–3.21)	0.89 (0.48–1.65)	0.96 (0.51–1.80)
**Place of Residence**								
Urban	1.00	1.00	1.00	1.00	1.00	1.00	1.00	1.00
Rural	1.42 (1.10–1.84) **	1.22 (0.92–1.61)	0.96 (0.53–1.73)	0.71 (0.37–1.37)	1.66 (0.94–2.93)	1.45 (0.78–2.68)	1.50 (1.07–2.11) *	1.33 (0.93–1.91)
**Island Group of Residence**								
Luzon	1.00	1.00	1.00	1.00	1.00	1.00	1.00	1.00
Visayas	1.24 (0.93–1.67)	1.08 (0.80–1.47)	1.40 (0.69–2.84)	1.16 (0.56–2.43)	1.48 (0.78–2.80)	1.28 (0.66–2.47)	1.14 (0.78–1.66)	0.98 (0.66–1.45)
Mindanao	0.93 (0.67–1.30)	0.78 (0.55–1.10)	1.40 (0.67–2.90)	1.05 (0.48–2.30)	1.32 (0.67–2.59)	1.22 (0.60–2.46)	0.70 (0.45–1.11)	0.61 (0.38–0.97) *
**Highest Educational Attainment**								
No Education, Preschool	1.00	1.00	1.00	1.00	1.00	1.00	1.00	1.00
Primary	0.69 (0.43–1.10)	0.60 (0.37–0.98) *	0.60 (0.23–1.54)	0.65 (0.24–1.76)	0.54 (0.22–1.34)	0.60 (0.23–1.57)	0.76 (0.38–1.52)	0.57 (0.28–1.17)
Secondary or Higher	0.55 (0.34–0.90) *	0.53 (0.31–0.92) *	0.61 (0.23–1.65)	0.83 (0.26–2.66)	0.43 (0.17–1.11)	0.60 (0.20–1.77)	0.56 (0.27–1.13)	0.43 (0.20–0.94) *
**Economic Status**								
Poor	1.00	1.00	1.00	1.00	1.00	1.00	1.00	1.00
Middle	1.27 (0.93–1.73)	1.37 (0.99–1.90)	0.78 (0.36–1.70)	0.69 (0.30–1.56)	0.87 (0.43–1.76)	1.03 (0.49–2.18)	1.66 (1.12–2.46) *	1.85 (1.22–2.81) **
Rich	0.67 (0.50–0.90) **	0.76 (0.53–1.09)	0.50 (0.26–0.99) *	0.35 (0.15–0.82) *	0.65 (0.35–1.18)	0.88 (0.42–1.84)	0.75 (0.51–1.10)	0.91 (0.58–1.45)
**Presence of Health Insurance**								
No	1.00	1.00	1.00	1.00	1.00	1.00	1.00	1.00
Yes	1.39 (1.03–1.88) *	1.55 (1.14–2.11) **	1.54 (0.85–2.80)	2.04 (1.07–3.89) *	0.75 (0.44–1.28)	0.82 (0.47–1.44)	1.74 (0.95–3.20)	1.93 (1.04–3.58) *
**Self–Reported NCDs ^b^**								
No	1.00	1.00	1.00	1.00	1.00	1.00	1.00	1.00
Yes	0.94 (0.61–1.45)	0.91 (0.59–1.41)	0.00 (0.00)	0.00 (0.00)	1.28 (0.61–2.72)	1.30 (0.60–2.82)	1.00 (0.58–1.72)	0.89 (0.51–1.55)

COR, crude odds ratio; AOR, adjusted odds ratio; CI, confidence interval; *** *p* value < 0.001; ** *p* value < 0.01; * *p* value < 0.05; ^a^ injury includes wounds, burns, or fractures; ^b^ non-communicable disease (NCDs) includes diabetes mellitus, cancer (all forms), or hypertension; the model was adjusted for all variables simultaneously.
